# Phase I drug-interaction study of effects of calcium and magnesium infusions on oxaliplatin pharmacokinetics and acute neurotoxicity in colorectal cancer patients

**DOI:** 10.1186/1471-2407-13-495

**Published:** 2013-10-25

**Authors:** Catherine H Han, Prashannata Khwaounjoo, Dean H Kilfoyle, Andrew Hill, Mark J McKeage

**Affiliations:** 1 Department of Pharmacology and Clinical Pharmacology and Auckland Cancer Society Research Centre, School of Medical Sciences, Faculty of Medical and Health Sciences, University of Auckland, Auckland, New Zealand; 2 Department of Medical Oncology, Auckland City Hospital, Auckland, New Zealand; 3 Department of Neurophysiology, Auckland City Hospital, Auckland, New Zealand

**Keywords:** Oxaliplatin, Calcium and magnesium, Neurotoxicity, Colorectal cancer, Hyperexcitability, Pharmacokinetics, Acute neuropathy

## Abstract

**Background:**

Calcium and magnesium (Ca/Mg) infusions have been suggested as an effective intervention for preventing oxaliplatin-induced neurotoxicity, but the effects of Ca/Mg infusions on oxaliplatin pharmacokinetics, motor nerve hyperexcitability and acute neurotoxicity symptoms are unclear.

**Methods:**

In this double blind crossover study, colorectal cancer patients undergoing oxaliplatin-based chemotherapy were randomised to receive Ca/Mg (1g Ca Gluconate plus 1g MgSO_4_) on cycle 1 and placebo (vehicle alone) on cycle 2, or to receive the same treatments in the opposite sequence. Study endpoints included plasma pharmacokinetics of intact oxaliplatin and free platinum; electromyography (EMG) detection of abnormal spontaneous high-frequency motor unit action potential discharges; and patient-reported acute neurotoxicity symptoms and their preferred study treatment for reducing these symptoms.

**Results:**

Nineteen of 20 enrolled patients completed the study. Plasma pharmacokinetics of intact oxaliplatin and free platinum were similar when oxaliplatin was given with Ca/Mg or placebo (ratio of geometric means of AUC_0-t_ with Ca/Mg or placebo: intact oxaliplatin, 0.95 (90% CI, 0.90 – 1.01); free platinum, 0.99 (90% CI, 0.94 – 1.05)). EMG motor nerve hyperexcitability scores were similar with Ca/Mg and placebo (mean difference in EMG score between Ca/Mg and placebo: -0.3 (95% CI, -2.2 – 1.6)). Patient-reported acute neurotoxicity symptoms were similar in frequency with Ca/Mg and placebo. For reducing neurotoxic symptoms, fewer patients preferred Ca/Mg than placebo or neither treatment (26% versus 74%; *P*<0.01).

**Conclusions:**

Ca/Mg infusions do not alter the clinical pharmacokinetics of oxaliplatin and do not seem to reduce its acute neurotoxicity.

**Trial registration:**

Trial registration identifier ACTRN12611000738921

## Background

Oxaliplatin-based chemotherapy has become an important treatment for gastrointestinal cancers, including colorectal, esophagogastric and pancreatic cancers [1-8]. In particular, it is a standard therapy for colorectal cancer in both the adjuvant and palliative settings [1,4-7]. Peripheral neurotoxicity is a major dose-limiting toxicity of oxaliplatin, which may compromise the delivery and full therapeutic potential of this drug to achieve tumour control or cure. Acute neurotoxicity occurs in a high proportion of patients shortly after oxaliplatin administration, but may resolve within a few hours or days, and is characterised by cold-related paresthesia, dysesthesia or allodynia, jaw stiffness, and muscle cramps [9]. These acute symptoms may reflect the induction of a state of acute peripheral nerve hyperexcitability detectable on electromyography (EMG) and other neurophysiological studies after oxaliplatin treatment [10-12]. Chronic peripheral sensory neurotoxicity from oxaliplatin can also be troublesome and is characterised by glove-and-stocking paresthesia and dysesthesia, and loss of peripheral deep tendon reflexes, vibration sensation and proprioception [9]. Its recovery after discontinuation of oxaliplatin may be slow, improving gradually over many months or even years, and is incomplete in some patients.

Calcium and magnesium (Ca/Mg) infusions were adopted by many oncologists in an attempt to reduce oxaliplatin-induced neurotoxicity based on the results of a retrospective analysis by Gamelin et al. [13] and subsequent reports [14]. However, there had been no consensus about the efficacy of Ca/Mg infusions for preventing oxaliplatin-induced neurotoxicity, and considerable variation existed in clinical practice with the use of this otherwise simple intervention. The mechanisms of the neuroprotective action of Ca/Mg infusions were also unknown. Several mechanisms have been postulated involving sodium or other ion channels [15], but these are unsubstantiated. We hypothesised that Ca/Mg infusions could reduce oxaliplatin neurotoxicity by altering the pharmacokinetics of oxaliplatin or by suppressing peripheral nerve hyperexcitability induced by oxaliplatin, possibly via an ion channel mechanism. Recently, we found that the conversion of oxaliplatin to its major degradation product, Pt(DACH)Cl_2_, is accelerated in the presence of calcium or magnesium ions in physiological chloride solutions *in vitro,* suggesting the potential for a pharmacokinetic interaction to exist between oxaliplatin and Ca/Mg infusions in cancer patients [16]. Previously, we had also developed techniques for detecting and measuring oxaliplatin-induced peripheral nerve hyperexcitability in cancer patients based on EMG [10], and for quantitating intact oxaliplatin and free platinum in human plasma samples [17], for application in this study.

In the current cross-over study, we aimed to determine the effect of Ca/Mg infusions on the pharmacokinetics of intact oxaliplatin and free platinum in colorectal cancer patients undergoing oxaliplatin-based chemotherapy. Pharmacokinetic studies were carried out on cycles one and two of oxaliplatin treatment using fully validated sample processing and bioanalytical techniques, based on high-performance liquid chromatography (HPLC) and inductively coupled plasma mass spectrometry (ICP-MS). The effects of Ca/Mg infusions on acute oxaliplatin-induced motor nerve hyperexcitability were previously unknown, but objectively evaluated in the current study using EMG techniques. Study patients reported their acute neurotoxicity symptoms and which study treatment they preferred for reducing these symptoms. To reduce bias, patients and researchers, including the neurophysiologist and bioanalyst, were blinded to study treatment details, and the study used a prospective design, placebo control and random assignment of the sequence of study treatments.

## Methods

### Patients

Eligible subjects were adult patients with colorectal cancer who were to receive standard chemotherapy with an oxaliplatin-based regimen (XELOX or mFOLFOX6) and who had given written informed consent. Subjects with pre-existing peripheral neuropathy, hypercalcemia, hypermagnesemia or medical contraindications to electromyography (EMG) or repeated pharmacokinetic blood sampling were ineligible. The study was approved by the Northern Y Regional Ethics Committee (Approval number NTY/11/01/005).

### Design

This was a prospective, randomised, double-blind, placebo-controlled, cross-over study. Patients were randomly allocated to receive either intravenous Ca/Mg (1g calcium gluconate plus 1g magnesium sulphate in 100 ml 5% dextrose) or placebo (100 ml 5% dextrose) over 15 minutes immediately before and after a two hour oxaliplatin infusion on cycle 1 then crossed over to the other study treatment on cycle 2. Randomisation was carried out by sealed envelope using 1:1 allocation, stratification for oxaliplatin dose (130 mg/m^2^ versus 85 mg/m^2^) and a block size of four. Double-blinding was achieved by neither the patients nor any of the research staff knowing the treatment assignment, except an independent oncology nurse who carried out the randomisation, made up the study infusion and recorded the treatment assignment. The active and placebo study treatments were made in identical 5% dextrose 100 ml infusion bags. Patients received standard chemotherapy and supportive care medications according to the standard protocols of our institution.

### Endpoints

The primary endpoint was plasma pharmacokinetic parameters of intact oxaliplatin, including area under the concentration versus time curve from time zero to the last sample (AUC_0-t_), mean residence time (MRT), clearance (Cl), peak plasma concentration (C_max_), and volume of distribution at steady state (V_ss_). The secondary endpoints included free platinum pharmacokinetic parameters, EMG detected abnormal spontaneous high-frequency motor unit action potentials, patient-reported neurotoxicity symptoms and study treatment preference for reducing neurotoxicity symptoms at the end of cycle 2.

### Pharmacokinetic procedures and analysis

On the day of treatment, blood samples were collected at each of the following time points: pre-infusion; at 20, 40, 60 and 90 minutes after the start of the oxaliplatin infusion; at the end of the infusion and 5, 10, 20, 30, 60, 120 and 180 minutes thereafter. As oxaliplatin is highly unstable in blood and plasma, blood samples were immediately processed to prepare methanol-deproteinised plasma, snap frozen in liquid nitrogen then stored at -80°C until analysis, under which conditions oxaliplatin remains stable [17]. Plasma concentrations of intact oxaliplatin and free platinum were determined by validated HPLC and ICP-MS method [17]. Calibration curve linearity (R^2^ > 0.99), accuracy (> 86%) and precision (< 13%), both within and between runs, fulfilled the requirements for validated bioanalytical assays [18]. Pharmacokinetic parameters were calculated using non-compartmental methods and PKSolver [19]. Pharmacokinetic analyses were undertaken with researchers blind to study treatment assignment.

### Neurophysiological assessment

An EMG was performed on day 2 of each study cycle to assess motor nerve hyperexcitability by a neurophysiologist who was blinded to the study treatment assignment. Standard EMG procedures were used and limb temperature was monitored and maintained above 32°C. EMG was performed in the first dorsal interosseous, extensor digitorum communis, tibialis anterior and gastrocnemius muscles. Motor unit activity was scored as in our previous study [10]:

0 No abnormal motor unit activity

1 increased insertional activity

2 spontaneous high frequency motor unit activity with muscle clinically at rest, with bursts lasting for duration of less than 2 seconds

3 spontaneous high frequency motor unit activity with muscle clinically at rest, with bursts lasting for duration of 2 to 5 seconds

4 spontaneous high frequency motor unit activity with muscle clinically at rest, with bursts lasting for duration of more than 5 seconds.

The final EMG score was calculated as the sum of scores from the four muscles tested on each cycle for each patient (minimum value, 0; maximum value, 16).

### Patient-reported neurotoxicity symptom evaluation

Patients completed a questionnaire after treatment cycles 1 and 2 to document the presence or absence of acute neurotoxicity symptoms (cold-induced paresthesia, jaw or throat tightness, pain at infusion site, paresthesia unrelated to cold, muscle cramps, change in vision, shortness of breath or other neurotoxicity symptoms). At the end of treatment cycle 2, patients were asked which of the two study treatments given for reducing neurotoxicity they preferred (cycle 1, cycle 2 or no preference).

### Statistical analysis

The statistical analysis of the primary endpoint was carried out in accordance with recommendations for the analysis of drug interaction studies by the US Food and Drug Administration [20]. As recommended, geometric mean and 90% confidence intervals (CI) of each pharmacokinetic parameter ratio for evaluable patients were calculated. If the 90% CIs for the geometric mean ratio fell within a no-effect boundary of between 80% and 125%, then it was to be concluded that there was no significant effect of Ca/Mg infusions on the pharmacokinetics of oxaliplatin. No additional dose-normalization was required as each pharmacokinetic parameter for each patient was expressed as ratio of that with Ca/Mg infusions versus placebo. Descriptive statistics were used to analyse the EMG data and patient-reported neurotoxicity outcomes. The statistical significance of differences in means and proportions between the placebo and Ca/Mg infusion groups were analysed by a paired t-test and Chi-square test, respectively. *P* values less than 0.05 were regarded as indicating statistical significance.

The study sample size was calculated based on the 90% CI for the geometric mean ratio of oxaliplatin pharmacokinetic parameters when oxaliplatin is given with and without Ca/Mg infusions, and coefficient of variations of oxaliplatin pharmacokinetic parameters of 25% or less. On this basis, a sample size of 12 evaluable patients was needed to define 90% CI for pharmacokinetic parameter ratio of ± 0.12 and for detecting a change in oxaliplatin pharmacokinetics of 25% or more with statistical significance. To allow for dropouts and discontinuations, a total of 20 patients were planned for enrolment.

## Results

### Patients and treatment

**Figure 1:**
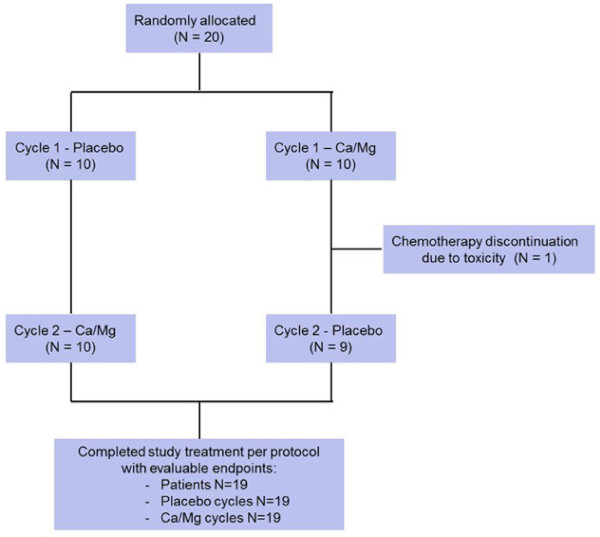
CONSORT diagram.

A total of 20 patients were enrolled between June 2011 and July 2012 who were to receive either XELOX (16 patients) or modified FOLFOX6 (4 patients) for colorectal adenocarcinoma. None of the patients had prior chemotherapy or abnormal serum calcium and magnesium levels. Baseline patient characteristics (Table 1) were well balanced between those randomly allocated to receive Ca/Mg infusions on the first cycle of chemotherapy then placebo infusion on the second treatment cycle, and those allocated to receive the same study treatments but in the opposite sequence. All patients completed the study except one patient who developed severe chemotherapy related enterocolitis and stopped chemotherapy after cycle one; therefore, this patient was not evaluable (Figure 1). A total of 38 treatment cycles was available for analysis (19 for placebo and 19 for Ca/Mg). All 19 patients completing the study received the same dose of oxaliplatin on both treatment cycles except one patient who had a 10% dose reduction for cycle 2 due to general intolerance of chemotherapy. There were no adverse effects attributable to the placebo or Ca/Mg infusions.

**Table 1:** Baseline clinical characteristics (n=20)

**Characteristic**	**No. of patients**	**%**
**Age**, years		
Median	62	
Range	31-77	
**Gender**		
Male	12	60
Female	8	40
**Ethnicity**		
European	15	75
Maori	3	15
Asian	2	10
**ECOG**^**1**^**status**		
0	14	70
1	6	30
**Tumor stage**		
2	1	5
3	11	55
4	8	40
**Chemotherapy regimen**		
XELOX^2^	16	80
modified FOLFOX6^3^	4	20
**Neurotoxicity risk factors**		
None	17	85
Diabetes	2	10
Spinal injury	1	5

^1^ECOG, Eastern Cooperative Oncology Group.

^2^XELOX, 2-hour infusion of oxaliplatin (130 mg/m^2^) followed by oral capecitabine (1250 mg/m^2^) twice daily day 1 to day 14 every 3 weeks.

^3^FOLFOX6, 2-hour infusion of oxaliplatin (85 mg/m^2^) together with 2-hour infusion of leucovorin (400 mg/m^2^) followed by a fluorouracil bolus (400 mg/m^2^) and 46-hour infusion (2,400 mg/m^2^) every 2 weeks.

### Pharmacokinetics

**Figure 2:**
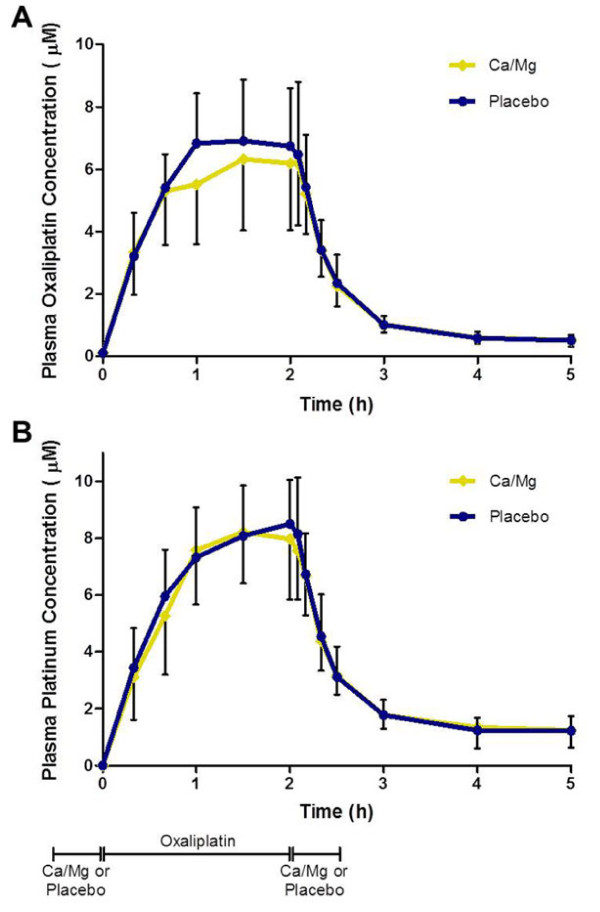
**Plasma concentration versus time curves.** For **(A)** intact oxaliplatin and **(B)** free platinum in colorectal cancer patients (n=19) given oxaliplatin with placebo (blue circle and line) or Ca/Mg infusions (gold diamond and line). Data points represent the geometric mean and bars the standard deviation. The horizontal line at the bottom of the figure represents the duration and sequence of study treatments.

Plasma concentrations versus time profiles of intact oxaliplatin (Figure 2A) and free platinum (Figure 2B) were similar when oxaliplatin was given with and without Ca/Mg infusions. Plasma concentrations of intact oxaliplatin and free platinum increased during the first hour of the oxaliplatin infusion, plateaued during the second hour, and then decreased rapidly after the end of the oxaliplatin infusion. The plasma concentration values for free platinum appeared to be similar or slightly higher than those for intact oxaliplatin at each time point.

Pharmacokinetic parameters of both intact oxaliplatin and free platinum were similar when oxaliplatin was given with Ca/Mg or placebo (Table 2). Ratios of geometric mean values of AUC_0-t_, C_max_, Cl, V_ss_ and MRT for Ca/Mg versus placebo ranged from 0.95 to 1.06 and their respective 90% CIs fell within the predefined no-effect boundaries of 0.8 to 1.25. AUC_0-t_ values for free platinum were approximately 30% higher than those for intact oxaliplatin. A subgroup of 15 patients given an oxaliplatin dose of 130 mg/m^2^, showed similar trends in pharmacokinetic parameter ratios and 90% CIs compared to the whole group, but their Cmax and AUC_0-t_ values for both intact oxaliplatin and free platinum were higher than for those given 85 mg/m^2^.

**Table 2:** Plasma pharmacokinetic parameters of intact oxaliplatin and free platinum in colorectal cancer patients (n=19)

			**AUC**_**0-t**_	**C**_**max**_	**Cl**	**V**_**ss**_	**MRT**
			(μmol/L*h)	**(μmol/L)**	**(L/h)**	**(L)**	**(h)**
**Oxaliplatin dose**		**Study treatment**					
All (n = 19)	Intact oxaliplatin	Placebo	15.6 (3.80)	7.62 (1.92)	33.6 (7.70)	57.2 (15.5)	1.68 (0.10)
		Ca/Mg	14.8 (3.69)	7.31 (1.91)	35.3 (9.76)	59.3 (14.9)	1.70 (0.13)
		Ratio*	0.95 (0.90-1.01)	0.96 (0.90-1.02)	1.05 (0.99-1.12)	1.06 (0.99-1.13)	1.01 (0.99-1.04)
	Free platinum	Placebo	20.2 (4.12)	9.24 (1.52)	25.9 (6.65)	48.4 (11.3)	1.87 (0.10)
		Ca/Mg	19.8 (4.18)	9.52 (1.55)	26.8 (6.03)	50.9 (9.95)	1.90 (0.16)
		Ratio*	0.98 (0.93-1.03)	1.03 (0.95-1.12)	1.04 (0.97-1.10)	1.05 (0.99-1.12)	1.02 (0.99-1.05)
130 mg/m^2^ (n = 15)	Intact oxaliplatin	Placebo	16.9 (3.32)	8.28 (1.69)	34.5 (7.98)	57.3 (14.3)	1.66 (0.07)
		Ca/Mg	15.8 (3.51)	8.01 (1.63)	36.7 (10.2)	60.7 (17.8)	1.66 (0.07)
		Ratio*	0.93 (0.86-1.02)	0.97 (0.90-1.04)	1.06 (0.98-1.15)	1.06 (0.98-1.14)	1.00 (0.98-1.02)
	Free platinum	Placebo	21.8 (3.09)	9.83 (1.06)	26.8 (6.57)	49.7 (11.7)	1.85 (0.05)
		Ca/Mg	21.3 (4.45)	10.0 (1.31)	27.8 (6.01)	52.2 (9.82)	1.88 (0.16)
		Ratio*	0.98 (0.92-1.04)	1.02 (0.94-1.10)	1.04 (0.97-1.12)	1.05 (0.97-1.14)	1.01 (0.98-1.05)
85 mg/m^2^ (n = 4)	Intact oxaliplatin	Placebo	11.4 (1.10)	5.58 (0.74)	30.7 (6.34)	53.8 (12.5)	1.75 (0.18)
		Ca/Mg	11.4 (1.03)	5.19 (0.53)	30.7 (5.81)	57.5 (10.3)	1.88 (0.16)
		Ratio*	1.00 (0.82-1.22)	0.93 (0.74-1.17)	1.00 (0.87-1.15)	1.07 (0.88-1.31)	1.07 (0.97-1.18)
	Free platinum	Placebo	15.3 (4.12)	7.32 (1.52)	22.9 (6.89)	43.9 (9.17)	1.92 (0.21)
		Ca/Mg	15.0 (2.79)	7.91 (1.35)	23.3 (5.17)	46.4 (10.4)	1.99 (0.17)
		Ratio*	0.98 (0.83-1.16)	1.08 (0.73-1.60)	1.02 (0.86-1.21)	1.06 (0.97-1.15)	1.03 (0.91-1.17)

Data values are the geometric mean (standard deviation). Ratio of the geometric mean values for Ca/Mg versus placebo were all close to unity and their 90% CIs all fell within predefined non-effect boundaries (0.8 to 1.25).

*Ratio of geometric mean (90% CI): with Ca/Mg / with placebo.

*Abbreviations*: AUC_0-t_, area under the curve from time 0 (before oxaliplatin dose) to the last concentration (5 hours from the start of the infusion); C_max_, maximum concentration; Cl, clearance; V_ss_, volume of distribution at steady state; MRT, mean residence time.

### Motor nerve hyperexcitability

**Figure 3:**
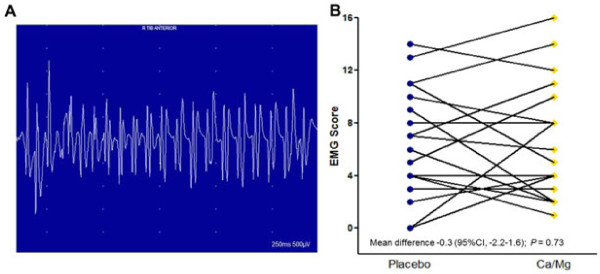
**Oxaliplatin-induced motor nerve hyperexcitability. (A)** Representative example of abnormal spontaneous high frequency motor unit action potentials detected on electromyography (EMG) on day 2 after oxaliplatin treatment (severity grade 4). **(B)** EMG motor nerve hyperexcitability score by study treatment on day 2 after treatment cycles one and two given with placebo (blue circle) or Ca/Mg (gold diamond) infusions. Individual scores for each patient are joined by the lines. EMG scores were calculated as described in the methods section.

EMGs were performed on Day 2 of treatment cycles one and two to assess motor nerve excitability. Abnormal spontaneous high frequency motor unit activity was detected in 19 of 19 patients (100%) given oxaliplatin with Ca/Mg and in 16 of 19 patients (84%) given oxaliplatin with placebo (Figure 3A). An EMG score was calculated from the severity of motor nerve hyperexcitability and the number of muscles affected. EMG motor nerve hyperexcitability score ranged from zero to 16 in different patient and treatment cycles (Figure 3B). The mean EMG score was 6.5 (SD, 4.31) with Ca/Mg and 6.2 (SD, 4.34) with placebo. The mean difference in EMG motor nerve hyperexcitability scores between placebo and Ca/Mg infusions for each patient was -0.3 (95% CI, -2.2 – 1.6).

### Patient-reported acute neurotoxicity symptoms and study treatment preference

**Figure 4:**
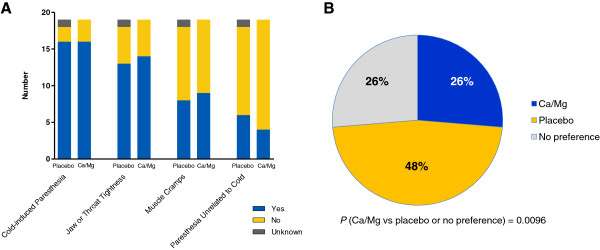
**Acute neurotoxicity. (A)** Patient reported acute neurotoxicity symptoms; and **(B)** study treatment preferences. The frequency of acute neurotoxicity symptoms was similar when oxaliplatin was given with Ca/Mg or placebo infusions. Fewer patients preferred Ca/Mg infusions for reducing their neurotoxicity symptoms compared to the number who preferred placebo or had no preference.

Patient-reported acute neurotoxicity symptoms were similar in frequency when oxaliplatin was given with placebo or Ca/Mg infusions (Figure 4A). Cold-induced paresthesia was reported by 16 of 19 patients (84%) given placebo and 16 of 19 patients (84%) given Ca/Mg infusions. Jaw or throat tightness was reported by 13 of 19 patients (68%) given placebo and 14 of 19 patients (74%) given Ca/Mg infusions. Muscle cramps were reported by 8 of 19 patients (42%) given placebo and 9 of 19 patients (47%) given Ca/Mg infusions. Few other acute neurotoxicity symptoms were reported. The preferred study treatment selected by patients for reducing neurotoxicity symptoms was placebo in 9 patients (47%), Ca/Mg in 5 patients (26%), and no preference in 5 patients (26%) (Figure 4B). Significantly fewer patients preferred Ca/Mg infusions for reducing their neurotoxicity symptoms than those who preferred placebo or neither treatment (26% versus 74%; *P* = 0.01).

## Discussion

The present study is the first, which we are aware of, undertaken to evaluate the effects of Ca/Mg infusions on the pharmacokinetics of intact oxaliplatin and motor nerve hyperexcitability. We showed that Ca/Mg infusions do not alter the pharmacokinetics of either intact oxaliplatin or free platinum, and our evidence also indicates that these infusions may provide no benefit in reducing acute oxaliplatin-induced neurotoxicity. The prospective, randomised, double-blind, placebo-controlled design of our study, together with the use of objective and patient-reported endpoints of acute neurotoxicity, eliminates potential sources of bias and provides reliable results.

We found no evidence of a pharmacokinetic interaction between Ca/Mg and oxaliplatin in our study. The plasma concentration versus time profiles and pharmacokinetic parameters of intact oxaliplatin and free platinum were similar when oxaliplatin was given with Ca/Mg or placebo infusions. Ratios of their pharmacokinetic parameters were close to unity and their respective 90% CIs fell within predefined no-effect boundaries of 0.8 and 1.25. We found intact oxaliplatin to be the major platinum species freely circulating in the plasma after oxaliplatin treatment, accounting for approximately 75% and 80% of the free platinum AUC_0-t_ and C_max_, respectively, as previously reported [17,21]. The only other study that we are aware of that attempted to compare the plasma pharmacokinetics of platinum in patients given oxaliplatin with or without Ca/Mg infusions was reported by Ishibashi *et al.*[22]. However, the plasma platinum concentration values reported in their study were much lower than expected and over 2000-times lower than those from our study. We suspect that loss of platinum analyte due to the instability of oxaliplatin and its reactivity with blood components during the delayed processing of blood samples may have contributed to invalid plasma platinum concentration measurements in their study, although it is not possible to be certain about this.

Most patients in our study demonstrated abnormal spontaneous high-frequency motor unit action potentials on EMGs undertaken about 24 hours after oxaliplatin, as previously reported by our group and others [10-12], but the frequency and severity of these EMG abnormalities did not reduce with the use of Ca/Mg infusions. Coinciding with this, similar proportion of patients reported acute neurotoxicity symptoms associated with oxaliplatin between the two treatment cycles given with or without Ca/Mg infusions, and fewer patients preferred Ca/Mg infusions than those preferring placebo or neither study treatment. No differences were apparent in EMG scores and patient reported neurotoxicity symptoms between those who received 130 mg/m^2^ and 85 mg/m^2^ of oxaliplatin, but this analysis was limited by small sample sizes of these subgroups. Although our study suggests that Ca/Mg infusions may have no effect on oxaliplatin neurotoxicity, it showed that repeated EMG assessment of motor hyperexcitability was feasible in patients receiving oxaliplatin chemotherapy. All patients complied with these procedures without loss of data in a total of 38 EMGs, thereby proving objective measurements, which complimented patient-reported endpoints of neurotoxicity. The mechanism of oxaliplatin-induced motor nerve hyperexcitability remains unclear but its neurophysiological features are reminiscent of neuromyotonia [12], and may reflect an acute state of generalised peripheral nerve hyperexcitability. If motor and sensory neuropathies are related in this way, future interventions identified to reduce EMG detectable motor nerve hyperexcitability may also have potential for preventing oxaliplatin-induced sensory neurotoxicity.

Our findings disagree with some previous reports regarding the clinical use of Ca/Mg infusions with oxaliplatin. A retrospective analysis by Gamelin et al. was the first to suggest that Ca/Mg infusions may reduce the neurotoxicity of oxaliplatin [13]. Then several prospective randomised trials [14,22-24] attempted to evaluate the neuroprotective effects of Ca/Mg infusions, but all except one trial [18] was prematurely terminated when concerns were raised about lowered tumour responses with Ca/Mg infusions. The retrospective design and early closures of subsequent trials may have introduced bias. Evaluation of cumulative chronic neurotoxicity of oxaliplatin was not possible in our cross-over study. However, the important question about the efficacy of Ca/Mg infusions in preventing chronic oxaliplatin neurotoxicity was addressed by Loprinzi *et al.* in their large prospective randomised placebo-controlled, double blind trial [25]. They found that Ca/Mg infusions do not reduce cumulative neuropathy of oxaliplatin as measured by the sensory scale of the EORTC QLQ-CIPN20 tool. Their findings are complimentary to ours, as both studies, despite their differing methodologies, demonstrated that Ca/Mg infusions are not effective in reducing oxaliplatin-induced neuropathy.

Potential limitations of this study include its modest sample size, single centre design and potential for carryover effects. The statistical power of this study was considered a priori when sample size calculations were undertaken using the pharmacokinetics of oxaliplatin as a primary endpoint. Subsequently, actual sample sizes and standard deviations of secondary neurotoxicity endpoints were also used for post-hoc power analyses. These analyses showed that with use of a crossover study design, a sample size of 19 or more evaluable patients has adequate statistical power (≥0.8) to detect fairly large but clinically meaningful changes in oxaliplatin pharmacokinetics (≥25%), EMG motor nerve hyperexcitability score (≥40%) and in the frequency of cold-induced paresthesia (≥38%), with statistical significance (≤0.05). No carry-over effects were evident between cycles one and two in the pharmacokinetics of intact oxaliplatin and free platinum or endpoints of neurotoxicity. Thus, the lack of change found in these endpoints with Ca/Mg infusions in the current study might be regarded as true negative findings. This highly efficient and feasible crossover design maybe applied for future initial clinical evaluation of other potentially promising approaches to preventing oxaliplatin neurotoxicity.

## Conclusions

Ca/Mg infusions do not alter the clinical pharmacokinetics of oxaliplatin and do not seem to reduce its acute neurotoxicity.

## Competing interests

The authors declare that they have no competing interests.

## Authors’ contributions

CHH coordinated patient recruitment and study procedures, and contributed to the pharmacokinetic sample analysis, data interpretation, statistical analysis and preparation of the final manuscript. PK contributed to pharmacokinetic sample analysis and data interpretation. DHK contributed to the study design and carried out the neurophysiological assessments. AH contributed to the study conception and design. MJM contributed to the study conception, design, data interpretation, statistical analysis and preparation of the final manuscript. All authors read and approved the final manuscript.

## Pre-publication history

The pre-publication history for this paper can be accessed here:

http://www.biomedcentral.com/1471-2407/13/495/prepub
